# The use of radiocobalt as a label improves imaging of EGFR using DOTA-conjugated Affibody molecule

**DOI:** 10.1038/s41598-017-05700-7

**Published:** 2017-07-20

**Authors:** Javad Garousi, Ken G. Andersson, Johan H. Dam, Birgitte B. Olsen, Bogdan Mitran, Anna Orlova, Jos Buijs, Stefan Ståhl, John Löfblom, Helge Thisgaard, Vladimir Tolmachev

**Affiliations:** 1 Department of Immunology, Genetics and Pathology, Uppsala University, Uppsala, Sweden; 2 Department of Protein Technology, KTH - Royal Institute of Technology, Stockholm, Sweden; 3 Department of Nuclear Medicine, Odense University Hospital, Sdr. Boulevard 29, 5000 Odense, Denmark; 4 Division of Molecular Imaging, Department of Medicinal Chemistry, Uppsala University, Uppsala, Sweden

## Abstract

Several anti-cancer therapies target the epidermal growth factor receptor (EGFR). Radionuclide imaging of EGFR expression in tumours may aid in selection of optimal cancer therapy. The ^111^In-labelled DOTA-conjugated Z_EGFR:2377_ Affibody molecule was successfully used for imaging of EGFR-expressing xenografts in mice. An optimal combination of radionuclide, chelator and targeting protein may further improve the contrast of radionuclide imaging. The aim of this study was to evaluate the targeting properties of radiocobalt-labelled DOTA-Z_EGFR:2377_. DOTA-Z_EGFR:2377_ was labelled with ^57^Co (T_1/2_ = 271.8 d), ^55^Co (T_1/2_ = 17.5 h), and, for comparison, with the positron-emitting radionuclide ^68^Ga (T_1/2_ = 67.6 min) with preserved specificity of binding to EGFR-expressing A431 cells. The long-lived cobalt radioisotope ^57^Co was used in animal studies. Both ^57^Co-DOTA-Z_EGFR:2377_ and ^68^Ga-DOTA-Z_EGFR:2377_ demonstrated EGFR-specific accumulation in A431 xenografts and EGFR-expressing tissues in mice. Tumour-to-organ ratios for the radiocobalt-labelled DOTA-Z_EGFR:2377_ were significantly higher than for the gallium-labelled counterpart already at 3 h after injection. Importantly, ^57^Co-DOTA-Z_EGFR:2377_ demonstrated a tumour-to-liver ratio of 3, which is 7-fold higher than the tumour-to-liver ratio for ^68^Ga-DOTA-Z_EGFR:2377_. The results of this study suggest that the positron-emitting cobalt isotope ^55^Co would be an optimal label for DOTA-Z_EGFR:2377_ and further development should concentrate on this radionuclide as a label.

## Introduction

A transmembrane epidermal growth factor receptor (EGFR) regulates cellular proliferation, motility and apoptosis^1^. Aberrant expression of EGFR has been detected in multiple cancers and linked to a malignant phenotype^2^. Overexpression of EGFR is a predictive biomarker for response of advanced non-small-cell lung cancer to tyrosine kinase inhibitor gefitinib^3, 4^ and to first-line chemotherapy plus anti-EGFR monoclonal antibody cetuximab^5^. In head and neck squamous cell carcinomas (HNSCC), overexpression of EGFR is associated with relapse after radiotherapy^6^ and can be used for selection of patients for hyperfractionated accelerated radiotherapy^7^. Preclinical studies suggest that determination of EGFR expression can be used for monitoring of response to combination of radiation therapy and cetuximab in HNSCC^8^ or to therapy using heat shock protein 90 inhibitors^9^. Radionuclide molecular imaging is a potential method for *in vivo* measurement of EGFR expression in malignant tumours. Unlike biopsy-based methods, molecular imaging is non-invasive and can be used repeatedly. In addition, radionuclide imaging enables imaging of receptor expression in multiple metastases addressing the heterogeneity of expression. Thus, the use of radionuclide molecular imaging of EGFR expression would make cancer treatment more personalized.

Approaches for development of probes for visualization of EGFR include the use of radiolabelled tyrosine kinase inhibitors, anti-EGFR monoclonal antibodies and their fragments, the natural ligand EGF and scaffold proteins^10^. A general challenge for development of EGFR-imaging agents is expression of EGFR in normal tissues, particularly in liver. Sequestering of radiolabelled probes in liver might result in their insufficient uptake in tumours^11^. However, a clinical study has demonstrated that it is possible to find an injected anti-EGFR antibody dose capable of providing a partial saturation of EGFR in the liver without target saturation in tumours enabling visualization of EGFR-expressing malignancies using radiolabelled antibodies^11^.

We develop EGFR-imaging probes based on Affibody molecules. Affibody molecules are small (7 kDa) engineered scaffold proteins, which can be selected by phage display for binding with high affinity to different proteins^12^. *In vivo* molecular imaging using radiolabelled Affibody molecules has been demonstrated in preclinical studies for several cancer-related molecular targets, e.g. HER2, EGFR, HER3, and IGF-1R. Clinical studies have demonstrated specific and high-contrast imaging of HER2-expression in metastatic breast cancer using a ^111^In- and ^68^Ga-labelled ABY-025 Affibody molecule^13, 14^. Earlier, we have shown that the anti-EGFR ^111^In-DOTA-Z_EGFR:2377_ Affibody molecule can visualize EGFR expression in murine xenografts^15^. Importantly, this Affibody molecule has similar affinity to both human and murine EGFR. According to surface plasmon resonance measurements, equilibrium dissociation constants (K_D_) were 0.8 and 28 nM for K_D1_ and K_D2_, respectively, in the case of murine EGFR. Corresponding values for human EGFR were 0.9 and 28 nM^15^. Therefore, a murine model takes into account the target-specific interaction of the tracer both in tumours and in normal tissues and should thus ensure good translational potential. Our studies showed that injection of 30–50 µg ^111^In-DOTA-Z_EGFR:2377_ provides partial saturation of EGFR in normal tissues without saturating the target in tumours^15^. Internalization of ^111^In-DOTA-Z_EGFR:2377_ by EGFR-expressing cells was slow, less than 20% after 24 h. Therefore, normal EGFR-expressing organs acted as depot that release ^111^In-DOTA-Z_EGFR:2377_ resulting in a relatively slow blood clearance. The tumour-to-blood ratios at optimal dosage were 7.0 ± 0.5 and 14 ± 4, at 4 and 24 h after injection, respectively.

A goal for the development of imaging agents is to obtain a probe with high uptake in tumour and low uptake in normal tissues. Earlier studies have demonstrated that different combinations of a chelator and a radionuclide can modify physico-chemical properties of a tumour-targeting peptide in different ways and influence its biodistribution^16, 17^. Moreover, the use of the same chelator in combination with different nuclides has resulted in altered imaging properties of somatostatin, RGD-peptides and bombesin analogues^17–22^. For example, Heppeler and co-workers concluded after comparison of ^57^Co- and ^68^Ga-labelled somatostatin analogue DOTA-TOC that “the physical features (size, charge) of the radiometal strongly influence receptor affinity, biodistribution and the pharmacokinetics of radiometal-labelled peptides”^22^. A strong influence of a radiometal/chelator combination on imaging contrast has been observed also for Affibody molecules. For example, biodistribution and tumour-to-organ ratios of DOTA-conjugated anti-HER2 Affibody molecules were altered appreciably by substitution of ^68^Ga by ^111^In or ^44^Sc and by substitution of ^111^In by ^57^Co^23–25^. Importantly, systematic studies concerning influence of radionuclides on biodistribution of tumour-targeting Affibody conjugates might help to select a probe with the optimal imaging characteristics.

Three cobalt radioisotopes, ^55^Co (T_1/2_ = 17.5 h), ^57^Co (T_1/2_ = 271.8 d) and ^58m^Co (T_1/2_ = 9.04 h) are of interest for radionuclide tumour targeting. ^55^Co is a long-lived positron emitter, which can be used for labelling of peptides for PET imaging^26, 27^. ^58m^Co emits Auger electrons and has been proposed for radionuclide therapy^26, 28^. The long-lived ^57^Co is a convenient surrogate radionuclide, which can be used for preclinical development of radiocobalt-labelled probes^22, 27^ in the same way as ^125^I is used as a substitute for ^123^I, ^124^I and ^131^I.

Development of a radiocobalt-labelled Z_EGFR:2377_ derivative with favourable targeting properties would open the way for the use of ^55^Co as a label. The increase of tumour-to-blood ratio of ^111^In-DOTA-Z_EGFR:2377_ with time indicates that performing the imaging at the next day after injection would provide better contrast. For this purpose, a positron-emitting radionuclide with a half-life of 10–20 h would be optimal. Besides, our studies on the previous generation of anti-EGFR Affibody molecules demonstrated that residualizing radiometal labels provide appreciably better tumour retention of the radionuclide at 24 h after injection^29^. ^55^Co can be produced by the ^54^Fe(*d*, *n*)^55^Co reaction on enriched ^54^Fe targets using low-energy cyclotrons available at PET-centres^26, 30^. Importantly, the DOTA chelator provides stable coupling of radiocobalt to targeting proteins and peptides^22, 25, 26, 28^. The use of a positron-emitting label for DOTA-ZEGFR:2377 would further improve detection of EGFR expression *in vivo*, since PET provides better imaging sensitivity compared with SPECT. A generator-produced positron emitter ^68^Ga (T_1/2_ = 67.7 min) is one obvious alternative to ^55^Co, although the time window for imaging would be limited to three-four hour after injection.

The goal of this study was to confirm that the DOTA-conjugated Z_EGFR:2377_ can be labelled with ^55^Co with preserved binding specificity and to compare tumour-targeting properties of radiogallium- and radiocobalt-labelled DOTA-Z_EGFR:2377._ The long-lived cobalt radioisotope ^57^Co (T_1/2_ = 271.8 d) was used in a number of experiments as a convenient surrogate of ^55^Co.

## Results

### Tracer production and labelling

The C-terminal cysteine-containing anti-EGFR Affibody molecule Z_EGFR:2377_ was produced, conjugated with maleimido-derivative of DOTA and purified as described earlier^15^. Analytic reversed-phase high-performance liquid chromatography (RP-HPLC) demonstrated a purity for DOTA-Z_EGFR:2377_ of over 95% (Supplementary Fig. S1). The molecular mass of DOTA-Z_EGFR:2377_ was determined by mass spectrometric analysis and showed a good agreement with the theoretical value (expected 7916.9 Da, found 7918 Da) (Supplementary Fig. S2). Measuring of circular dichroism spectra (Supplementary Fig. S3) demonstrated a high-fidelity refolding of DOTA-Z_EGFR:2377_ after heating up to 90 °C.

Labelling of DOTA-Z_EGFR:2377_ with cobalt isotopes provided yields of over 99%. The identity of conjugates was confirmed by radio-SDS-PAGE. Initial experiments concerning labelling with ^55^Co were associated with precipitation of Affibody molecules. Exclusion of glassware from the radionuclide production solved this problem. Still, aggregation of ^55^Co-DOTA-Z_EGFR:2377_ remains to be an issue. Similar effect for ^57^Co-DOTA-Z_EGFR:237_ was observed only occasionally, e.g. in the case of prolonged (3 h) incubation at 85 °C. Remarkably, this phenomenon was observed only with Affibody molecules, but not during ^55^Co-labelling of somatostatin or bombesin analogues^26–28^. Labelling of DOTA-Z_EGFR:2377_ with ^68^Ga provided a yield of 98.9 ± 0.5%. A purification using NAP-5 column provided a purity of 100%. A summary of obtained yields and specific activities of imaging probes is provided in Supplementary Table S1. There was no measurable release of radionuclide from the conjugate after 4 h (^57^Co) or 2 h (^68^Ga) incubation with 500-fold excess of EDTA.

### *In vitro* characterization of radiolabelled DOTA-Z_EGFR:2377_

**Figure 1:**
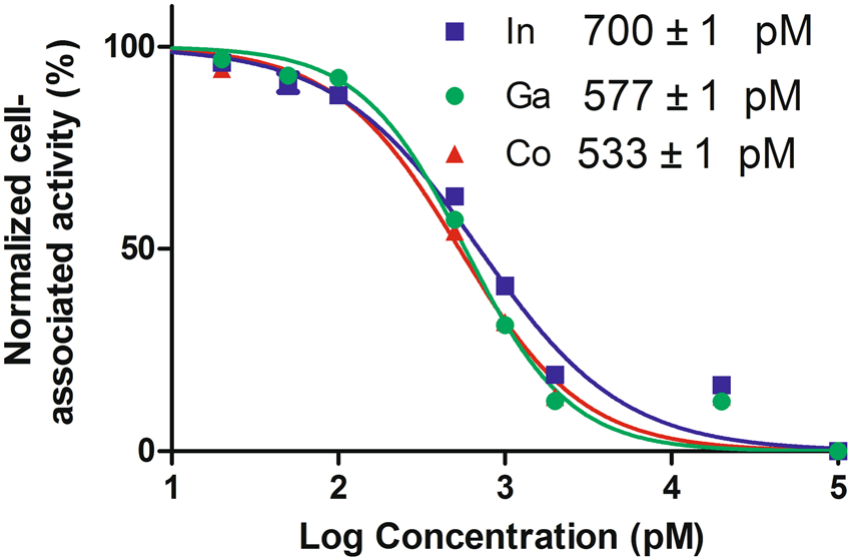
Inhibition of ^111^In-DOTA- Z_EGFR:2377_ binding to A431 cells with ^nat^Ga-, ^nat^Co- or ^nat^In- Z_EGFR:2377_. Data presented as mean ± SD of 3 culture dishes.

Pre-treatment of EGFR-expressing A431 cells with an excess of non-labelled Z_EGFR:2377_ caused a more than fivefold reduction of binding of both ^68^Ga- and ^55/57^Co- DOTA-Z_EGFR:2377_ (p < 5 × 10^−7^). This demonstrates saturable receptor-mediated binding of all conjugates to EGFR-expressing cells. According to LigandTracer measurements, the equilibrium dissociation constant (K_D_) of ^57^Co-labelled DOTA-Z_EGFR:2377_ interaction with A431 cells (167 ± 18 pM) was lower than K_D_ of ^111^In-DOTA-Z_EGFR:2377_ (744 ± 28 pM) (For sensorgrams see Supplementary Fig. S4). Both labelled variants had similar association rate, (4.8 ± 0.6) × 10^4^ M^−1^s^−1^ and (3.7 ± 0.1) × 10^4^ M^−1^s^−1^, for ^57^Co- DOTA-Z_EGFR:2377_ and ^111^In-DOTA-Z_EGFR:2377_, respectively. However, the dissociation rate for ^57^Co- DOTA-Z_EGFR:2377_, (7.8 ± 0.7) × 10^−6^ s^−1^, was appreciably lower than for ^111^In-DOTA-Z_EGFR:2377_, (2.8 ± 0.2) × 10^−5^ s^−1^. Unfortunately, K_D_ of ^68^Ga-DOTA-Z_EGFR:2377_ could not be measured since the half-life of the radionuclide is much shorter than the assay time required for accurate determination of the off-rate. To evaluate the relative binding strength of not only cobalt and indium but also gallium-labelled variants, the half maximum inhibition concentrations (IC_50_) were measured (Fig. 1). In agreement with K_D_ determination, the IC_50_ value of Co-DOTA-Z_EGFR:2377_ (533 ± 1pM) was lower than that of In-DOTA-Z_EGFR:2377_ (700 ± 1pM). IC_50_ values of Co-DOTA-Z_EGFR:2377_ and Ga-DOTA-Z_EGFR:2377_ (576 ± 1pM) were very close, but significantly different.

**Figure 2:**
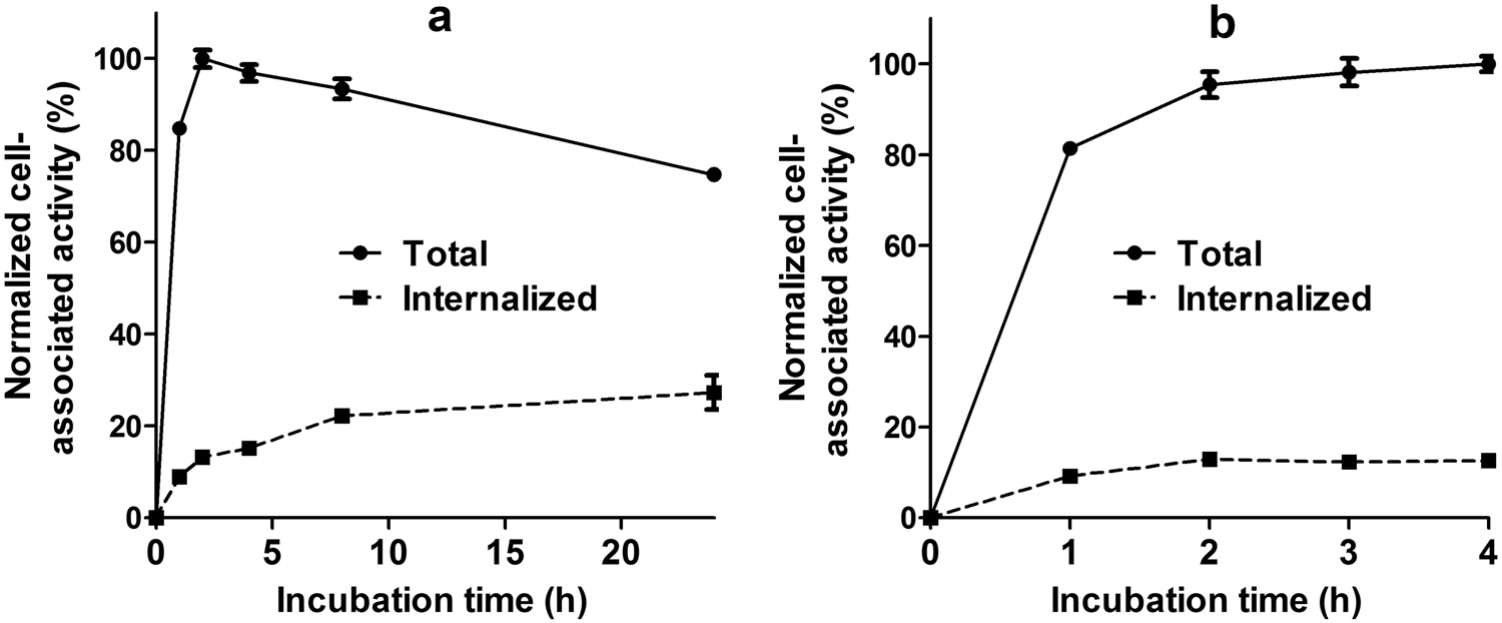
Cell-associated radioactivity as a function of time during continuous incubation of EGFR-expressing A431 cells with the ^57^Co-DOTA-Z_EGFR:2377_ (**a**) and ^68^Ga- DOTA-Z_EGFR:2377_ (**b**). Data are presented as mean from 3 dishes ± SD, and normalized to the maximum uptake. Due to small variability some error bars are hidden behind the symbols.

Data concerning internalization of ^57^Co-DOTA-Z_EGFR:2377_ and ^68^Ga-DOTA-Z_EGFR:2377_ by EGFR-expressing A431 cells are presented in Fig. 2. In agreement with previous data for ^111^In-DOTA-Z_EGFR:2377_^15^, the internalization was slow, although the internalized fraction of the radioactivity increased over time. After 4-h incubation, the internalized fraction was less than 20% for both tracers. In the case of ^57^Co-DOTA-Z_EGFR:2377_, the cell-associated radioactivity reached maximum between 2 and 4 h with subsequent decline.

### *In vivo* studies

**Figure 3:**
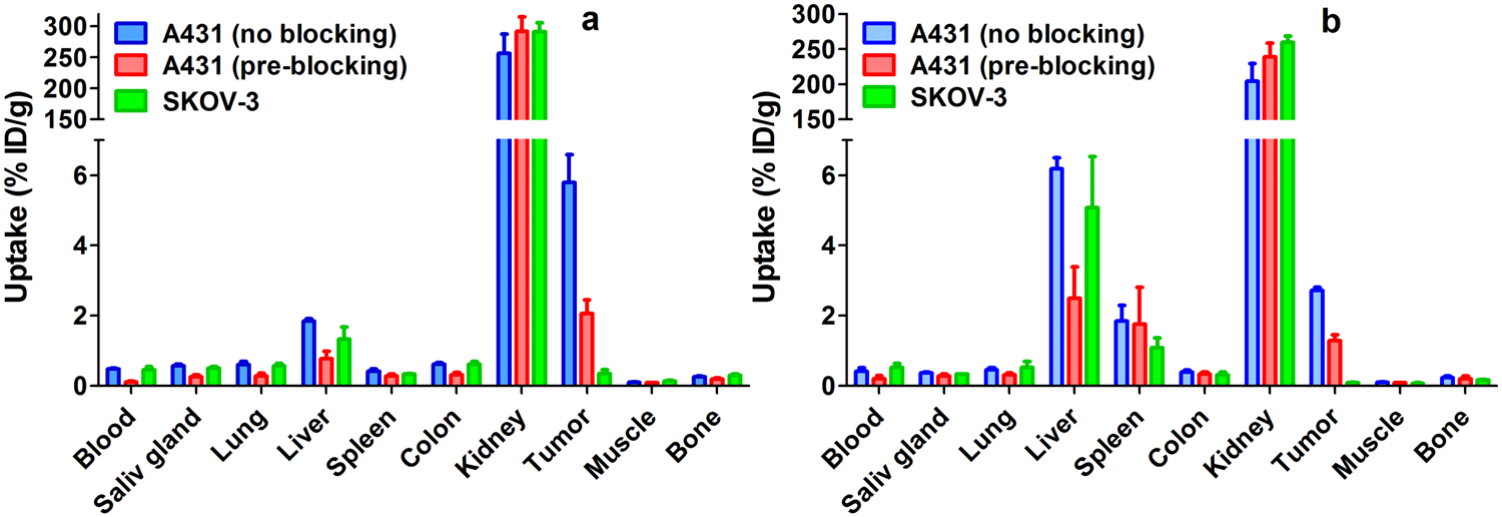
Specificity of ^57^Co-DOTA-Z_EGFR:2377_ (**a**) and ^68^Ga-DOTA-Z_EGFR:2377_ (**b**) uptake in tumours at 3 h after injection. An injected protein dose was adjusted to 40 µg for all animals. For pre-blocking, a group of animals bearing EGFR-positive A431 xenografts was pre-injected with 400 µg non-labelled Z_EGFR:2377_ 40 min before injection of the radiolabelled conjugate. A group with SKOV-3 xenografts was used as a negative control. Data are presented as average %ID/g ± SD (n = 4).

Specificity of EGFR targeting *in vivo* was evaluated by pre-saturation of the receptors in normal murine tissues and A431 xenografts with high EGFR expression by pre-injection of a 10-fold excess of non-labelled Z_EGFR:2377_. In addition, mice bearing SKOV-3 xenografts with very low EGFR expression were used as negative control. The results of the *in vivo* specificity experiments are presented in Fig. 3. Pre-saturation of EGFR in A431 xenografts caused a significant (p < 0.0005) decrease in tumour uptake of both tracers. Besides, there was a statistically significant (p < 0.05) decrease in radioactivity concentration in blood and lung, and in EGFR-expressing tissues, such as salivary gland, liver, and colon. The uptake of both ^57^Co-DOTA-Z_EGFR:2377_ and ^68^Ga-DOTA-Z_EGFR:2377_ in SKOV-3 xenografts was much lower (p < 10^−7^) than in A431 xenografts.

Comparison of biodistribution of ^68^Ga-DOTA-Z_EGFR:2377_ and ^57^Co-DOTA-Z_EGFR:2377_ is presented in Table 1. The most prominent difference was between ^68^Ga-DOTA-Z_EGFR:2377_ and ^57^Co-DOTA-Z_EGFR:2377_ in hepatic, splenic and tumour uptake. The uptake of ^68^Ga-DOTA-Z_EGFR:2377_ was 3.3-fold higher than the uptake of ^57^Co-DOTA-Z_EGFR:2377_ in liver, and 4.4-fold higher in spleen. The tumour uptake of ^57^Co-DOTA-Z_EGFR:2377_ was 2.1-fold higher than the uptake of ^68^Ga-DOTA-Z_EGFR:2377_. Besides, the uptake of ^57^Co-DOTA-Z_EGFR:2377_ was 1.2–1.5 fold higher in salivary gland, lung, and colon. At 24 h after injection, the uptake of ^57^Co-DOTA-Z_EGFR:2377_ was reduced 1.3–1.6 fold in tumour as well as in salivary gland, lung, and liver.

**Table 1:** Comparative biodistribution of ^68^Ga-DOTA-Z_EGFR:2377_ and ^57^Co-DOTA-Z_EGFR:2377_ after injection of 40 μg into BALB/C nu/nu mice bearing A431 xenografts.

	3 h after injection		24 h after injection
	^68^Ga-DOTA-Z_EGFR:2377_	^57^Co-DOTA-Z_EGFR:2377_	^57^Co-DOTA-Z_EGFR:2377_
Blood	0.42 ± 0.09	0.48 ± 0.02	0.13 ± 0.03^b^
Salivary gland	0.37 ± 0.02	0.57 ± 0.04^a^	0.40 ± 0.03^b^
Lung	0.46 ± 0.05	0.60 ± 0.09^a^	0.37 ± 0.04^b^
Liver	6.2 ± 0.3	1.85 ± 0.06^a^	1.2 ± 0.1^b^
Spleen	1.9 ± 0.4	0.42 ± 0.06^a^	0.38 ± 0.04
Colon	0.39 ± 0.05	0.61 ± 0.05^a^	0.47 ± 0.07^b^
Kidney	204 ± 25	256 ± 30^a^	218 ± 12
Tumour	2.7 ± 0.1	5.8 ± 0.8^a^	4.04 ± 0.03^b^
Muscle	0.10 ± 0.01	0.10 ± 0.01	0.09 ± 0.01
Bone	0.23 ± 0.04	0.25 ± 0.03	0.21 ± 0.05
Gastrointestinal tract	0.9 ± 0.1	1.1 ± 0.2	0.7 ± 0.2^b^
Carcass	3.8 ± 0.5	4.9 ± 0.4^a^	3.37 ± 0.08^b^

Data are presented as average %ID/g ± SD (n = 4), except for the gastrointestinal tract and carcass which are presented as %ID per sample.

^a^Significant (p < 0.05) difference between uptake of ^68^Ga-DOTA-Z_EGFR:2377_ and ^57^Co-DOTA-Z_EGFR:2377;_

^b^Significant (p < 0.05) difference between uptake of ^57^Co-DOTA-Z_EGFR:2377_ at 3 and 24 h after injection.

The difference in the distribution profile resulted in significantly (p < 0.05) higher tumour-to-organ ratios of ^57^Co-DOTA-Z_EGFR:2377_ compared to ^68^Ga-DOTA-Z_EGFR:2377_ for all measured organs and tissues at 3 h after injection (Table 2). The most stunning was the difference in liver, where the use of ^57^Co-DOTA-Z_EGFR:2377_ provided a positive contrast (tumour-to-liver ratio of 3.1 ± 0.5), while the tumour-to-liver ratio of ^68^Ga-DOTA-Z_EGFR:2377_ was below one. At 24 h after injection, the tumour-to-blood ratio for ^57^Co-DOTA-Z_EGFR:2377_ increased 2.6-fold, to 32 ± 7. There was minor but significant decrease of tumour-to-spleen and tumour-to-kidney ratios.

**Table 2:** Tumour-to-organ ratios of ^68^Ga-DOTA-Z_EGFR:2377_ and ^57^Co-DOTA-Z_EGFR:2377_ after injection of 40 μg into BALB/C nu/nu mice bearing A431 xenografts.

	3 h after injection		24 h after injection
	^68^Ga-DOTA-Z_EGFR:2377_	^57^Co-DOTA-Z_EGFR:2377_	^57^Co-DOTA-Z_EGFR:2377_
Blood	7 ± 2^a^	12 ± 2	32 ± 7^b^
Salivary gland	7.3 ± 0.4^a^	10 ± 2	10.2 ± 0.8
Lung	5.9 ± 0.7^a^	10 ± 2	11 ± 1
Liver	0.44 ± 0.03^a^	3.1 ± 0.5	3.3 ± 0.3
Spleen	1.5 ± 0.4^a^	13.9 ± 0.7	11 ± 1^b^
Colon	7.1 ± 0.8^a^	10 ± 1	9 ± 1
Kidney	0.013 ± 0.002^a^	0.023 ± 0.002	0.019 ± 0.001^b^
Muscle	26 ± 3^a^	56 ± 10	48 ± 7
Bone	12 ± 2^a^	24 ± 5	21 ± 5

Data are average of four animals ± SD.

^a^Significant (p < 0.05) difference between ^68^Ga-DOTA-Z_EGFR:2377_ and ^57^Co-DOTA-Z_EGFR:2377_.

^b^Significant (p < 0.05) difference between values for ^57^Co-DOTA-Z_EGFR:2377_ at 3 and 24 h after injection.

**Figure 4:**
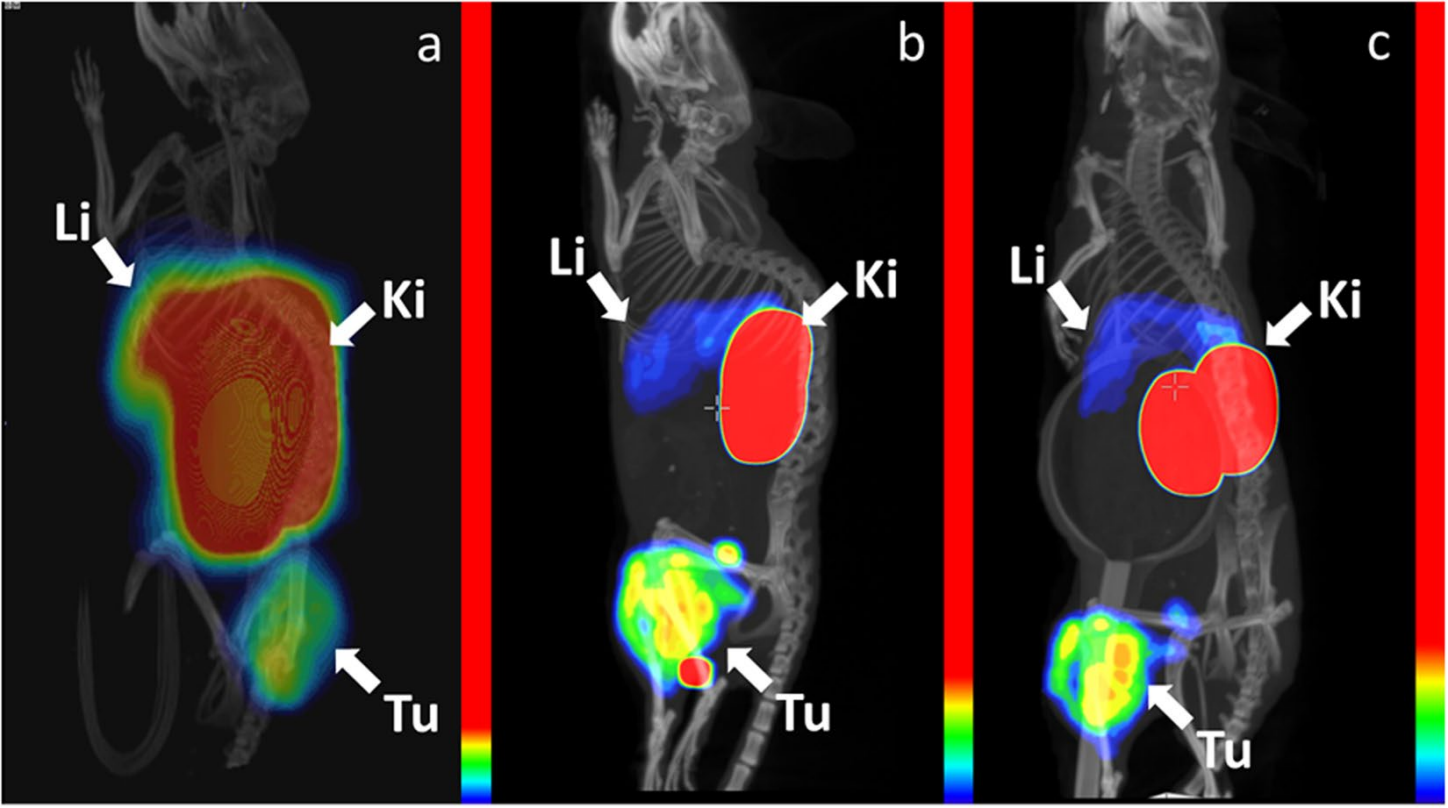
Imaging of mice bearing A431 xenografts. PET/CT using ^68^Ga-DOTA-Z_EGFR:2377_ at 3 h after injection (a) and SPECT/CT using ^57^Co-DOTA-Z_EGFR:2377_ at 3 h (**b**) and 24 h (**c**) after injection. The images show three-dimensional volume rendering of PET or SPECT data overlaid CT data. The images were rotated to provide view of liver. The relative colour scales were normalized to the highest activity and then adjusted to provide first red pixel in tumours (8, 13 and 17% of the full scale, for Fig. 4a,b and c, respectively). Arrows point at tumours (Tu), kidneys (Ki) and livers (Li).

Imaging using SPECT/CT and PET/CT devices for small animals, performed 3 h after injection (Fig. 4, full-scale image in Supplementary Fig. S5), confirmed the results of the biodistribution experiments. Kidneys were the organs with the highest uptake for both radionuclides. ^68^Ga-DOTA-Z_EGFR:2377_ provided a high contrast of A431 xenografts, even though the hepatic uptake was higher than the tumour uptake. On the other hand, the tumour uptake of ^57^Co-DOTA-Z_EGFR:2377_ was higher than the hepatic uptake.

## Discussion

Anti-EGFR DOTA-Z_EGFR:2377_ Affibody molecule was successfully labelled with the nuclides ^68^Ga and ^55/57^Co. Both gallium- and cobalt-loaded DOTA-Z_EGFR:2377_ had subnanomolar IC_50_ values (Fig. 1). ^57^Co-DOTA-Z_EGFR:2377_ and ^68^Ga-DOTA-Z_EGFR:2377_ showed specific binding to EGFR-expressing cells *in vitro* and *in vivo* (Fig. 3). Cellular processing of both ^57^Co-DOTA-Z_EGFR:2377_ and ^68^Ga-DOTA-Z_EGFR:2377_ was characterized by slow internalization (Fig. 2). Both conjugates enabled imaging of EGFR-expressing xenografts *in vivo* (Fig. 4). However, there was a striking difference in their biodistribution profile (Table 2). A common feature was a prominent hepatic uptake. The biodistribution profile of ^68^Ga-DOTA-Z_EGFR:2377_ was quite similar to the profile reported for ^111^In-DOTA-Z_EGFR:2377_^15^. The hepatic uptake was much higher than uptake in tumour or EGFR-expressing tissues, i.e. colon and salivary gland. In the case of ^57^Co-DOTA-Z_EGFR:2377_, the hepatic uptake was 3.4-fold lower and the tumour uptake was 2-fold higher compared to ^68^Ga-DOTA-Z_EGFR:2377_ at 3 h after injection of the same protein dose. This resulted in significantly higher tumour-to-organ ratios for all measured tissues. Most importantly, the tumour uptake of ^57^Co-DOTA-Z_EGFR:2377_ was significantly higher than the hepatic uptake (tumour-to-liver ratio of 3.1 ± 0.5), which is a precondition for successful imaging of hepatic metastases. This is essential, as liver is the major metastatic site for many cancers. The tumour-to-bone ratio for ^57^Co-DOTA-Z_EGFR:2377_ was also twice as high as for ^68^Ga-DOTA-Z_EGFR:2377_. At 24 h after injection, the tumour-to-blood ratio for ^57^Co-DOTA-Z_EGFR:2377_ increased 2.7-fold compared with 3 h time point.

The exact nature of this phenomenon is not quite clear. An important insight is provided by the fact that hepatic uptake of ^68^Ga-DOTA-Z_EGFR:2377_ was three-fold higher than uptake of ^57^Co-DOTA-Z_EGFR:2377_ (Fig. 3), when a blocking dose of non-labelled Z_EGFR:2377_ was pre-injected. Data for ^111^In-DOTA-Z_EGFR:2377_ show that saturation of liver uptake by non-labelled Z_EGFR:2377_ was much less efficient than saturation of uptake in other EGFR-expressing tissues^15^. This suggests that there are at least two different mechanisms of DOTA-Z_EGFR:2377_ hepatic uptake: one is dependent on EGFR expression (and can be saturated) and one is independent. The use of ^57^Co-DOTA-Z_EGFR:2377_ might reduce the hepatic uptake by the EGFR-independent mechanism. This is quite surprising, as the difference between ^57^Co-DOTA-Z_EGFR:2377_ and ^68^Ga-DOTA-Z_EGFR:2377_ is very subtle. Both Co^2+^ and Ga^3+^ have a six-coordination sphere and form DOTA complexes with distorted octahedral coordination geometry^22^. The major difference between Co-DOTA and Ga-DOTA complexes is that the cobalt complex carries a negative charge while the gallium complex is neutral. The literature data suggest that such small difference might have dramatic effect on affinity of radiolabelled peptides. For example, affinity of ^68^Ga and ^57^Co-DOTATOC to somatostatin receptors differs by one order of magnitude^22^. It is conceivable that the complex charge could also have an appreciable effect on DOTA-Z_EGFR:2377_ affinity to “scavenger receptors” in liver. The reduced liver binding of ^57^Co-DOTA-Z_EGFR:2377_ makes it more available for binding to EGFR-expressing tumours and tissues, which is in agreement with experimental observations.

The findings of this study suggest that the positron-emitting ^55^Co (T_1/2_ = 17.5 h) might be a suitable label for DOTA-Z_EGFR:2377_ and further development should concentrate on this radionuclide as a label. The half-life is long enough for imaging at 24 h after injection, but is not exceedingly long. The use of a positron-emitting label for DOTA-Z_EGFR:2377_ would further improve detection of EGFR expression *in vivo*, since PET provides better imaging sensitivity compared with SPECT.

Earlier, several approaches for preclinical development of PET probes specific to the extracellular domain of EGFR were evaluated. Labelling of the natural ligand, EGF, with ^68^Ga^31^ or ^18^F ^32^ enabled specific imaging of EGFR-expressing xenografts with tumour-to-blood ratios in the range of 2–5. At low doses, both tracers had liver uptake exceeding tumour uptake several fold. In mice, the hepatic uptake has been reduced appreciably at higher injected protein doses. However, this approach is not feasible clinically because of adverse side effects associated with injection of high doses of EGF (nausea, vomiting, hypotension)^33^. Anti-EGFR therapeutic monoclonal antibodies cetuximab and panitumumab were labelled with long-lived positron emitters ^64^Cu (T_1/2_ = 12.7 h), ^86^Y (T_1/2_ = 14.7 h), and ^89^Zr (T_1/2_ = 78.4 h)^34–39^. The maximum tumour-to-blood ratio (range of 2–8) was obtained at 3–4 days after injection due to slow clearance of antibodies from blood. ^57^Co-DOTA-Z_EGFR:2377_ provided a higher tumour-to-blood ratio already at 3 h after injection than any of these antibody-based agents at the best imaging time point. Labelling and preclinical *in vivo* evaluation of another clone of anti-EGFR Affibody molecule, Z_EGFR:1907_, with ^18^F and ^64^Cu has been reported^40–42^. Z_EGFR:1907_ had an apparent cross-reactivity with murine EGFR, which made the murine model adequate. These probes provided tumour-to-blood ratios of 2–10, depending on the labelling approach. Still, tumour-to-liver ratios were around 1.4 at best. Recently, we performed pre-clinical evaluation of Z_EGFR:2377_, which was site-specifically labelled with a long-lived positron emitter ^89^Zr (T_1/2_ = 78.4 h) using a maleimido derivative of a deferoxamine (DFO) chelator^43^. Direct comparison with ^89^Zr-labelled anti-EGFR antibody cetuximab demonstrated that ^89^Zr-DFO-Z _EGFR:2377_ provided appreciably higher tumour-to-blood, tumour-to-liver, and tumour-to-bone ratios. Yet, ^57^Co-DOTA-Z_EGFR:2377_ provided 2-5-fold higher tumour-to-organ ratios compared to ^89^Zr-DFO- _EGFR:2377_. Taken together, these data indicate that the radiocobalt-labelled DOTA-Z_EGFR:2377_ demonstrated the best tumour-to-non-tumour ratios among all pre-clinically tested affibody-based agents for imaging of EGFR.

A clinical translation of the finding of this study would require considering the dosimetry difference between ^68^Ga and ^55^Co. Although the longer half-life of ^55^Co permits a substantial improvement of tumour-to-organ ratios, it results in longer residence time and, therefore, higher dose burden on patients, compared to ^68^Ga. Another issue is that the decay scheme of ^55^Co includes a number of gamma emissions besides positrons, which increases whole-body dose (See Supplementary Table S2). These features are a common concern for all long-lived positron emitters. Still, the much more long-lived positron emitter ^89^Zr (T_1/2_ = 78.4 h) is used to label antibodies for clinical imaging^44^, and clinical studies show acceptable absorbed dose levels^45, 46^. It has to be noted that ^55^Co provides a better ratio between numbers of annihilation photons and co-emitted gamma photons compared to ^89^Zr and the majority of other long-lived positron emitters (Supplementary Table S2). Compared to the long-lived positron emitter ^64^Cu (T_1/2_ = 12.7 h), ^55^Co has higher positron abundance and does not emit beta particles. ^55^Co also provides a better image quality compared to the long-lived positron-emitting radiometal ^86^Y (T_1/2_ = 14.7 h)^47^.

In conclusion, the chemical nature of a radionuclide can appreciable influence the biodistribution and targeting properties of Affibody molecules. *In vivo* studies using ^57^Co as a model nuclide suggest that radiocobalt-labelled DOTA-Z_EGFR:2377_ is a very promising tracer for imaging of EGFR expression *in vivo*. Labelling of DOTA-Z_EGFR:2377_ with ^55^Co providing a conjugate with preserved specificity of binding to EGFR-expressing cells is feasible. This justifies further preclinical development of ^55^Co-labelled Affibody molecules for PET-imaging of EGFR expression in malignant tumours. Future studies should concentrate on development of large-scale production of ^55^Co-DOTA-Z_EGFR:2377_ and quantitative PET imaging of xenografts with different EGFR expression levels.

## Methods

### General

Maleimido-mono-amide-DOTA (1,4,7,10-tetraazacyclododecane-1,4,7-tris-acetic acid-10-maleimidoethylacetamide) was purchased from Macrocyclics (Dallas, TX). [^57^Co] cobalt chloride was purchased from PerkinElmer Sweden, (Upplands Väsby, Sweden). [^111^In] indium chloride was purchased from Mallinckrodt Pharmaceuticals (Chesterfield, United Kingdom). The EGFR-rich squamous carcinoma cell line A431 was obtained from American Type Culture Collection (ATCC, Rockville, MD). Buffers, used for conjugation and labelling, were purified from free metals using Chelex 100 resin (Bio-Rad Laboratories, Hercules, CA, USA).

The ^68^Ge/^68^Ga generator (50 mCi) was from Eckert and Ziegler (Vienna, Austria). Hydrochloric acid (0.1 M, prepared from 30% ultrapure HCl from Merck) was used for fractionated elution of the generator. The fraction containing the maximum radioactivity was used for labelling.

Labelling yield and radiochemical purity of the labelled DOTA-Z_EGFR:2377_ molecule were determined by radio-ITLC using 150–771 DARK GREEN strips (Biodex Medical Systems, New York, NY, USA) eluted with 0.2 M citric acid. In this system, free ^68^Ga and ^55/57^Co migrate with the solvent front and the radiolabelled DOTA-Z_EGFR:2377_ stays at the application point. The radio-ITLC was cross-validated by SDS-PAGE. The distribution of radioactivity along the thin layer chromatography strips and SDS-PAGE gels was measured using a Cyclone Storage Phosphor System (PerkinElmer, Wellesey, MA, USA).

Ketalar (ketamine, 50 mg/mL, Pfizer), Rompun (xylazin, 20 mg/mL, Bayer) and heparin (5000 IE/mL, Leo Pharma) were obtained commercially. The euthanasia was performed by an intra-peritoneal injection of Ketalar-Rompun solution (20 µL of solution/g body weight: Ketalar, 10 mg/mL; Rompun, 1 mg/mL).

The radioactivity uptake in the cellular processing and the biodistribution studies was measured using an automated gamma-counter with 3 inch NaI(TI) detector (1480 WIZARD, Wallac, Turku).

### Statistics

Data on cellular uptake and biodistribution were analysed by unpaired, two-tailed t-test using GraphPad Prism (version 4.00 for Windows GraphPad Software) in order to determine significant differences (p < 0.05).

### Tracer production and labelling

The C-terminal cysteine-containing anti-EGFR Affibody molecule Z_egfr:2377_ was produced, conjugated with maleimido-derivative of DOTA and purified as described earlier^15^. Before conjugation, a solution of Affibody molecules was treated with dithiothreitol (DTT; E. Merck, Darmstadt, Germany) in order to reduce spontaneously formed intermolecular disulfide bonds. For this purpose, a stock solution of Affibody molecules (2 ml, 2.3 mg/ml in PBS) was mixed with 100 µl 1 M Tris-HCl buffer, pH 8.0, and 63 µl DTT solution (0.5 M in water). The mixture was incubated at 40 °C for 30 min. The reduced Affibody molecules were then purified and the buffer was changed according to the manufacturer’s instructions using a disposable PD-10 column (GE Healthcare) pre-equilibrated with 0.2 M sodium acetate, pH 5.5. Maleimido-mono-amide-DOTA (2 mg) was added and the mixture incubated for 1 h at 40 °C.

The Affibody molecule was purified and analysed by high-performance liquid chromatography and on-line mass spectrometry (HPLC-MS) using an Agilent 1100 LC/MSD system equipped with electrospray ionization and a single quadrupole (Agilent Technologies, Palo Alto, CA). For purification, a Zorbax 300SB C18 9.4 × 250 mm, 5 µm column was used. The analysis was performed using a Zorbax 300SB-C18 2.1 × 150 mm, 3.5 µm column. Solvent A comprised 0.1% trifluoroacetic acid (TFA) in water, and solvent B comprised 0.1% TFA in acetonitrile. The column oven temperature was set to 30 °C. For analysis, the column was eluted with a linear gradient of 35% to 60% solvent B over 18 min, with a flow rate of 0.5 ml/min. The purified conjugate (further designated as DOTA-Z_EGFR:2377_) was freeze-dried. Chemstation Rev. B.02.01 software (Agilent) was used for analysis and evaluation of HPLC data. The conjugate was aliquoted (50 µg/aliquot) and lyophilized.

The thermal stability and secondary structure of DOTA- Z_EGFR:2377_ were determined using a JASCO J-810 spectropolarimeter (JASCO, Tokyo, Japan). The samples were diluted in PBS to concentrations of 0.5 mg/mL and melting temperatures were assessed by measuring the ellipticity at 221 nm during a temperature gradient ranging from 25–90 °C. The secondary structural content was assessed by measuring the degree of ellipticity from 250 to 195 nm at 25 °C before and after the thermal stability measurement.

^55^Co for labelling of Affibody molecules was produced via the ^54^Fe(d,n)^55^Co nuclear reaction as described earlier^26^. Briefly, the electroplated ^54^Fe target (99.84% enriched, Campro Scientific) was irradiated by 8.5 MeV deuterons using a GE PETtrace cyclotron. After the irradiation the produced ^55^Co was separated from the target material on an anion exchange column (Dowex 1∙8) using 4 M hydrochloric acid as eluent and further purified using a Chromafix 30-PS-HCO_3_ cartridge^28^ before evaporation to dryness. Labelling with radiocobalt has been performed according to previously described method^25^. Briefly, the lyophilized DOTA-Z_EGFR:2377_ (50 µg) was reconstituted in 40 µL of 0.2 M ammonium acetate, pH 5.5, mixed with radiocobalt stock solution (for ^57^Co: up to 18 µL, 4–15 MBq; for ^55^Co: 30–50 µL, 21–46 MBq) and incubated at 60°C for 30 min. The conjugate was purified using a NAP-5 size-exclusion column equilibrated with PBS.

For labelling with ^68^Ga, the ^68^Ge/^68^Ga generator was eluted with 0.1 M hydrochloric acid as eluent, and fractions containing 500 µL were collected. The fraction containing the maximum radioactivity was used for labelling. The lyophilized DOTA-Z_EGFR:2377_ (50 µg) was reconstituted in 100 µL 1.25 M sodium acetate buffer, pH 3.6. ^68^Ga-containing eluate, up to 3 MBq per microgram of DOTA-Z_EGFR:2377_, was added to the mixture. The mixture was incubated at 95 °C for 15 min. Thereafter, 1000-fold excess of Na_4_EDTA was added, and the mixture was incubated at 95 °C for 10 min. After labelling, the conjugate was purified using a NAP-5 size-exclusion column equilibrated with PBS.

Labelling of DOTA-Z_EGFR:2377_ with ^111^In for affinity measurements using LigandTracer and for determination of the half maximum inhibition concentration (IC_50_) was performed as described earlier^15^. For IC_50_ measurements, the conjugates were loaded with cobalt, indium and gallium of natural isotopic composition using the same conditions as for radiolabelling.

The yield and radiochemical purity of the conjugates were evaluated using ITLC eluted with 0.2 M citric acid, pH 2.0. To cross-validate the ITLC data, a radio-SDS page analysis was performed. The samples were treated with sample buffer (10 min at 70 °C) and analyzed using SDS-PAGE (at 200 V using NuPAGE 4–12% Bis-Tris Gels (Invitrogen AB, Sweden) in MES buffer (Invitrogen AB, Sweden). To provide a marker for low-molecular weight radioactivity, a sample of ^68^Ga or ^57^Co acetate was applied next to the analytes on the same gel.

To evaluate stability of labelling, the labelled conjugates were incubated with 500-fold excess of EDTA for 2 h at room temperature and then analysed using ITLC.

### *In vitro* characterization of radiolabelled DOTA-Z_EGFR:2377_

The EGFR-expressing A431 epidermoid carcinoma cell line (ATCC) was used. Ovarian carcinoma SKOV-3 cell line (ATCC) with very low EGFR expression was used as a negative control in *in vivo* studies. Cells were cultured using Ham’s F10-medium supplemented with 10% foetal bovine serum (Flow, Stockholm, Sweden), L-glutamine (2 mM) and PEST (penicillin 100 IU/mL and streptomycin 100 mg/mL).

The *in vitro* binding specificity was verified by receptor pre-saturation with unlabelled Affibody molecules as described^15^. To test the binding specificity, 8 nM of radiolabelled DOTA-Z_EGFR:2377_ was added to 6 petri dishes containing 7 × 10^5^ A431 cells/dish. A 50-fold molar excess of non-labelled DOTA-Z_EGFR:2377_ was added to groups of three petri dishes 15 min before the labelled conjugate to saturate the receptors. The dishes were incubated at 37 °C for 1 h in a humidified incubator. The media was collected, the cells were detached using trypsin-EDTA solution and radioactivity was measured. Percent of cell-bound radioactivity was calculated for both the pre-saturated and unsaturated cells.

The dissociation constant (K_D_) of ^111^In and ^57^Co-labelled DOTA-Z_EGFR:2377_ binding to living A431 cells was measured by using LigandTracer Yellow (Ridgeview Instruments AB, Uppsala, Sweden) and analysed using InteractionMap software (Ridgeview Diagnostics AB, Uppsala, Sweden) as described earlier^48^. This device records kinetic binding and dissociation of radiolabelled tracers on living cells. In order to cover the concentration span needed for proper affinity estimation, three increasing concentrations of the radioconjugate (0.33, 1, and 3 nM) were added in each affinity assay. Analysis was performed in duplicates.

To evaluate the relative binding strength of gallium, cobalt and indium-labelled Z_EGFR:2377_, the half maximum inhibition concentration (IC_50_) were measured using ^111^In-DOTA- Z_EGFR:2377_. Monolayers of A431 cells were incubated for 4 h at 4 °C with ^nat^Ga-, ^nat^Co- or ^nat^In-DOTA-Z_EGFR:2377_ (0–100 nM) in the presence of 10 nM ^111^In-DOTA- Z_EGFR:2377_. After incubation, the cells were washed with media and detached with trypsin. The detached cells were collected, and the cell-associated radioactivity was measured. The IC_50_ values were determined using GraphPad Prism software.

The rate of internalization of ^68^Ga-DOTA-Z_EGFR:2377_ and ^57^Co-DOTA-Z_EGFR:2377_ by EGFR-expressing A431 squamous carcinoma cell during continuous incubation was studied using the acid wash method^49^. The labelled compound (protein concentration of 8 nM) was added to 15 petri dishes containing approximately 7 × 10^5^ cells/dish. The cells were incubated at 37 °C in a humidified incubator. At pre-determined time points (1, 2, 3, and 4 h after incubation start for ^68^Ga-DOTA-Z_EGFR:2377_; 1, 2, 4, 8 and 24 h for ^57^Co-DOTA-Z_EGFR:2377_), the medium from a set of three dishes was removed. The cells were washed with 1 mL of ice-cold serum free medium. To collect the membrane-bound radioactivity, the cells were treated with 0.5 mL of 0.2 M glycine buffer containing 4 M urea, pH 2.0, for 5 min on ice. Dishes were washed with 0.5 mL acidic buffer followed by 1 mL PBS, and the fractions were pooled. To collect radioactivity internalized by the cells, treatment with 0.5 mL of 1 M NaOH at 37 °C for 30 min was performed. Dishes were additionally washed with 0.5 mL NaOH solution followed by 1 mL PBS, and the alkaline fractions were pooled. The percentage of internalized radioactivity was calculated for each time point.

### *In vivo* studies

The animal experiments were planned and performed in accordance with the national regulation on laboratory animals’ protection and were approved by the Ethics Committee for Animal Research in Uppsala. Euthanasia was performed under Rompun/Ketalar anaesthesia. Female BALB/c nu/nu mice were purchased from Taconic M&B (Ry, Denmark). At the time of experiment, the animals were 10 weeks old and the average weight was 21.3 ± 0.8 g. EGFR-expressing xenografts were established by subcutaneous injection of 10^7^ A431 cells in the right hind leg. The tumours were grown for 10–14 d. At the time of the study, the average tumour weight was 0.8 ± 0.3 g. The animals were randomized into groups of four. To test if the tumour uptake depends on EGFR expression, an additional group of mice was implanted with 10^7^ SKOV-3 cells having very low EGFR expression. A biodistribution experiment was performed 14 days after implantation. At the time of the study, the average animal weight was 19.7 ± 0.8 g and the average tumour weight was 0.16 ± 0.03 g.

For biodistribution measurements, one group of mice was intravenously injected with ^68^Ga-DOTA-Z_EGFR:2377_ (700 kBq in 100 µL PBS per mouse) and two groups with ^57^Co-DOTA-Z_EGFR:2377_ (30 kBq in 100 µL PBS per mouse). The injected protein dose was adjusted to 40 µg/mouse by non-labelled DOTA-Z_EGFR:2377_. The animals were sacrificed at 3 h after injection of ^68^Ga-DOTA-Z_EGFR:2377_ and at 3 and 24 h after injection of ^57^Co-DOTA-Z_EGFR:2377_. Blood and organ samples were collected and weighed, and their radioactivity was measured. Tissue uptake was calculated as percent of injected dose per gram (% ID/g). To check the specificity of *in vivo* EGFR targeting, groups of four mice were subcutaneously pre-injected with 400 μg non-labelled recombinant Z_HER2:342_ affibody molecule before injecting 40 μg ^68^Ga-DOTA-Z_EGFR:2377_ or ^57^Co-DOTA-Z_EGFR:2377_. The control groups were sacrificed at 3 h p.i. A group of mice bearing SKOV-3 xenografts with low EGFR expression was injected with a mixture of ^57^Co-DOTA-Z_EGFR:2377_ (10 kBq per mouse) and ^68^Ga-DOTA-Z_EGFR:2377_ (700 kBq per mouse). The injected protein dose was adjusted to 40 µg/mouse by non-labelled DOTA-Z_EGFR:2377_. These mice were euthanized at 3 h after injection. Spectra of each sample and a standard were recorded. For each samples, radioactivity of ^57^Co and ^68^Ga were calculated by integration of counts in the energy range 10–160 keV and 300–160 keV, respectively. The data were corrected for background, gamma spectrometer dead time for each sample, ^68^Ga decay during measurement and spillover of ^68^Ga counts into ^57^Co energy window.

Imaging was performed to obtain a visual confirmation of the biodistribution data. Mice bearing A431 xenografts were injected with 7 MBq ^68^Ga-DOTA-Z_EGFR:2377_ or 9 MBq ^57^Co-DOTA-Z_EGFR:2377_. The injected protein dose was 40 µg/mouse. PET imaging was performed at 3 h after injection using Triumph™ Trimodality system (Gamma Medica) PET/CT device for small rodents. The CT scan was performed at the following parameters: field of view (FOV), 8 cm; magnification, 1.48; one frame and 512 projections for 2.13 min. PET data were acquired in list mode during 30 min and reconstructed using OSEM-3D. CT raw files were reconstructed by filter back projection (FBP). SPECT/CT imaging was performed using nanoScan SC (Mediso Medical Imaging Systems, Hungary) at 3 and 24 h after injection at the following parameters: CT-energy peak of 50 keV, 670 μA, 480 projections, 2.29 min acquisition time. Helical SPECT acquisition was performed using ^57^Co energy window (109.89 keV–134.31 keV), 110 projection and matrix of 256 × 256. The time per projection was 32 sec (totally 60 min) for imaging at 3 h and 65 sec (totally 12 min) for imaging at 24 h. CT raw files were reconstructed using Nucline 2.03 Software (Mediso Medical Imaging Systems, Hungary). SPECT raw data were reconstructed using Tera-Tomo™ 3D SPECT reconstruction technology.

## Electronic supplementary material

Supplementary info

## References

[1] Avraham R, Yarden Y (2011). Feedback regulation of EGFR signalling: decision making by early and delayed loops. Nat. Rev. Mol. Cell Biol..

[2] Carlsson, J. EGFR-Family Expression and Implications for Targeted Radionuclide Therapy in *Targeted Radionuclide Tumor Therapy*-*Biological Aspects* (ed. Stigbrand, T. *et al*.) 25–58 (Springer, 2008).

[3] Cappuzzo F (2005). Epidermal growth factor receptor gene and protein and gefitinib sensitivity in non-small-cell lung cancer. J. Natl. Cancer Inst..

[4] Hirsch FR (2006). Molecular predictors of outcome with gefitinib in a phase III placebo-controlled study in advanced non-small-cell lung cancer. J. Clin. Oncol..

[5] Pirker R (2012). EGFR expression as a predictor of survival for first-line chemotherapy plus cetuximab in patients with advanced non-small-cell lung cancer: analysis of data from the phase 3 FLEX study. Lancet. Oncol..

[6] Ang KK (2002). Impact of epidermal growth factor receptor expression on survival and pattern of relapse in patients with advanced head and neck carcinoma. Cancer Res..

[7] Bentzen SM (2005). Epidermal growth factor receptor expression in pretreatment biopsies from head and neck squamous cell carcinoma as a predictive factor for a benefit from accelerated radiation therapy in a randomized controlled trial. J. Clin. Oncol..

[8] van Dijk LK, Boerman OC, Franssen GM, Kaanders JH, Bussink J (2015). 111In-cetuximab-F(ab’)2 SPECT and 18F-FDG PET for prediction and response monitoring of combined-modality treatment of human head and neck carcinomas in a mouse model. J. Nucl. Med..

[9] Niu G, Cai W, Chen K, Chen X (2008). Non-invasive PET imaging of EGFR degradation induced by a heat shock protein 90 inhibitor. Mol. Imaging Biol..

[10] Corcoran EB, Hanson RN (2014). Imaging EGFR and HER2 by PET and SPECT: a Review. Med. Res. Rev..

[11] Divgi CR (1991). Phase I and imaging trial of indium 111-labeled anti-epidermal growth factor receptor monoclonal antibody 225 in patients with squamous cell lung carcinoma. J. Natl. Cancer Inst..

[12] Ahlgren. S, Tolmachev V (2010). Radionuclide molecular imaging using Affibody molecules. Curr. Pharm. Biotechnol..

[13] Sörensen J (2014). First-in-human molecular imaging of HER2 expression in breast cancer metastases using the 111In-ABY-025 affibody molecule. J. Nucl. Med..

[14] Sörensen J (2016). Measuring HER2-Receptor Expression In Metastatic Breast Cancer Using [68Ga]ABY-025 Affibody PET/CT. Theranostics.

[15] Tolmachev V (2010). Imaging of EGFR expression in murine xenografts using site-specifically labelled anti-EGFR 111In-DOTA-Z EGFR:2377 Affibody molecule: aspect of the injected tracer amount. Eur. J. Nucl. Med. Mol. Imaging.

[16] Tolmachev V, Orlova A (2010). Influence of labelling methods on biodistribution and imaging properties of radiolabelled peptides for visualisation of molecular therapeutic targets. Curr. Med. Chem..

[17] Eisenwiener KP (2002). NODAGATOC, a new chelator-coupled somatostatin analogue labeled with [67/68Ga] and [111In] for SPECT, PET, and targeted therapeutic applications of somatostatin receptor (hsst2) expressing tumors. Bioconjug. Chem..

[18] Antunes P (2007). Are radiogallium-labelled DOTA-conjugated somatostatin analogues superior to those labelled with other radiometals?. Eur. J. Nucl. Med. Mol. Imaging..

[19] Decristoforo C (2008). 68Ga- and 111In-labelled DOTA-RGD peptides for imaging of alphavbeta3 integrin expression. Eur. J. Nucl. Med. Mol. Imaging..

[20] Dumont RA (2011). Novel (64)Cu- and (68)Ga-labeled RGD conjugates show improved PET imaging of α(ν)β(3) integrin expression and facile radiosynthesis. J. Nucl. Med..

[21] Varasteh Z (2013). Synthesis and characterization of a high-affinity NOTA-conjugated bombesin antagonist for GRPR-targeted tumor imaging. Bioconjug. Chem..

[22] Heppeler A (2008). Metal-ion-dependent biological properties of a chelator-derived somatostatin analogue for tumour targeting. Chemistry.

[23] Honarvar, H. *et al*. Position for site-specific attachment of a DOTA chelator to synthetic affibody molecules has a different influence on the targeting properties of 68Ga- compared to 111in-labeled conjugates. *Mol*. *Imaging* **13** (2014). 10.2310/7290.2014.00034 25249017

[24] Honavar H (2016). Evaluation of the first 44Sc-labeled Affibody molecule for imaging of HER2-expressing tumors. Nucl. Med. Biol..

[25] Wållberg H, Ahlgren S, Widström C, Orlova A (2010). Evaluation of the radiocobalt-labeled [MMA-DOTA-Cys61]-Z HER2:2395(-Cys) affibody molecule for targeting of HER2-expressing tumors. Mol. Imaging Biol..

[26] Thisgaard H, Olesen ML, Dam JH (2011). Radiosynthesis of ^55^Co- and ^58m^Co-labelled DOTATOC for positron emission tomography imaging and targeted radionuclide therapy. J. Label. Compd. Radiopharm.

[27] Dam JH, Olsen BB, Baun C, Høilund-Carlsen PF, Thisgaard H (2016). *In Vivo* Evaluation of a Bombesin Analogue Labeled with Ga-68 and Co-55/57. Mol. Imaging. Biol..

[28] Thisgaard H (2014). F. Evaluation of cobalt-labeled octreotide analogs for molecular imaging and auger electron-based radionuclide therapy. J. Nucl. Med..

[29] Tolmachev V (2009). Affibody molecules for epidermal growth factor receptor targeting *in vivo*: aspects of dimerization and labeling chemistry. J. Nucl. Med..

[30] Zaman MR, Spellerberg S, Qaim SM (2003). Production of 55Co via the 54Fe(d, n)-process and excitation functions of 54Fe(d, t)53Fe and 54Fe(d, α)52mMn reactions from threshold up to 13.8MeV. Radiochim. Acta.

[31] Velikyan I (2005). Preparation and evaluation of (68)Ga-DOTA-hEGF for visualization of EGFR expression in malignant tumors. J. Nucl. Med..

[32] Li W (2012). PET imaging of EGF receptors using [18F]FBEM-EGF in a head and neck squamous cell carcinoma model. Eur. J. Nucl. Med. Mol. Imaging..

[33] Cuartero-Plaza A (1996). Radiolocalization of squamous lung carcinoma with 131I-labeled epidermal growth factor. Clin. Cancer Res..

[34] Cai W (2007). Quantitative PET of EGFR expression in xenograft-bearing mice using 64Cu labeled cetuximab, a chimeric anti-EGFR monoclonal antibody. Eur. J. Nucl. Med. Mol. Imaging.

[35] Li WP, Meyer LA, Capretto DA, Sherman CD, Anderson CJ (2008). Receptor-binding, biodistribution, and metabolism studies of 64Cu-DOTAcetuximab, a PET-imaging agent for epidermal growth-factor receptor-positive tumors. Cancer Biother. Radiopharm..

[36] Nayak TK (2010). PET imaging of HER1-expressing xenografts in mice with 86Y-CHX-A”-DTPA-cetuximab. Eur. J. Nucl. Med. Mol. Imaging.

[37] Nayak TK, Garmestani K, Baidoo KE, Milenic DE, Brechbiel MW (2010). Preparation, biological evaluation, and pharmacokinetics of the human anti-HER1 monoclonal antibody panitumumab labeled with 86Y for quantitative PET of carcinoma. J. Nucl. Med..

[38] Aerts HJ (2009). Disparity between *in vivo* EGFR expression and 89Zr-labeled cetuximab uptake assessed with PET. J. Nucl. Med..

[39] Chang AJ, De Silva RA, Lapi SE (2013). Development and characterization of 89Zr-labeled panitumumab for immuno-positron emission tomographic imaging of the epidermal growth factor receptor. Mol. Imaging.

[40] Miao Z, Ren G, Liu H, Jiang L, Cheng Z (2010). Small-animal PET imaging of human epidermal growth factor receptor positive tumor with a 64Cu labeled affibody protein. Bioconjug. Chem..

[41] Miao Z (2012). PET of EGFR expression with an 18F-labeled affibody molecule. J. Nucl. Med..

[42] Su X (2014). Comparison of two site-specifically (18)F-labeled affibodies for PET imaging of EGFR positive tumors. Mol. Pharm..

[43] Garousi J (2016). PET imaging of epidermal growth factor receptor expression in tumours using 89Zr-labelled ZEGFR:2377 affibody molecules. Int. J. Oncol..

[44] Jauw YW (2016). Immuno-Positron Emission Tomography with Zirconium-89-Labeled Monoclonal Antibodies in Oncology: What Can We Learn from Initial Clinical Trials?. Front. Pharmacol.

[45] Makris NE (2015). PET/CT-derived whole-body and bone marrow dosimetry of 89Zr-cetuximab. J. Nucl. Med..

[46] Laforest R (2016). [89Zr]Trastuzumab: Evaluation of Radiation Dosimetry, Safety, and Optimal Imaging Parameters in Women with HER2-Positive Breast Cancer. Mol. Imaging. Biol..

[47] Braad PE, Hansen SB, Thisgaard H, Høilund-Carlsen PF (2015). PET imaging with the non-pure positron emitters: (55)Co, (86)Y and (124)I. Phys. Med. Biol..

[48] Barta P (2012). Protein interactions with HER-family receptors can have different characteristics depending on the hosting cell line. Int. J. Oncol..

[49] Wållberg H, Orlova A (2008). Slow internalization of anti-HER2 synthetic affibody monomer 111In-DOTA-ZHER2:342-pep2: implications for development of labeled tracers. Cancer Biother. Radiopharm.

