# Supplementary material for: The use of radiocobalt as a label improves imaging of EGFR using DOTA-conjugated Affibody molecule
Source: Sci Rep. 2017 Jul 20;7:5961. doi: 10.1038/s41598-017-05700-7 (PMC5519605; doi:10.1038/s41598-017-05700-7)
Supplement: Supplementary file 1 — Supplementary info [file 41598_2017_5700_MOESM1_ESM.pdf]

SUPPLEMENTARY INFORMATION

The use of radiocobalt as a label improves imaging of EGFR using DOTA-conjugated Affibody molecule

Javad Garousi[1], Ken G. Andersson[2], Johan H. Dam[3], Birgitte B. Olsen[3], Bogdan Mitran [4], Anna Orlova[4], Jos Buijs[1], Stefan Ståhl[2], John Löfblom[2], Helge Thisgaard[3], Vladimir Tolmachev [1*].

[1] Department of Immunology, Genetics and Pathology, Uppsala University, Uppsala, Sweden;

[2] Department of Protein Technology, KTH - Royal Institute of Technology, Stockholm, Sweden;

[3] Department of Nuclear Medicine, Odense University Hospital, Sdr. Boulevard 29, 5000 Odense, Denmark;

[4]Division of Molecular Imaging, Department of Medicinal Chemistry, Uppsala University, Uppsala, Sweden

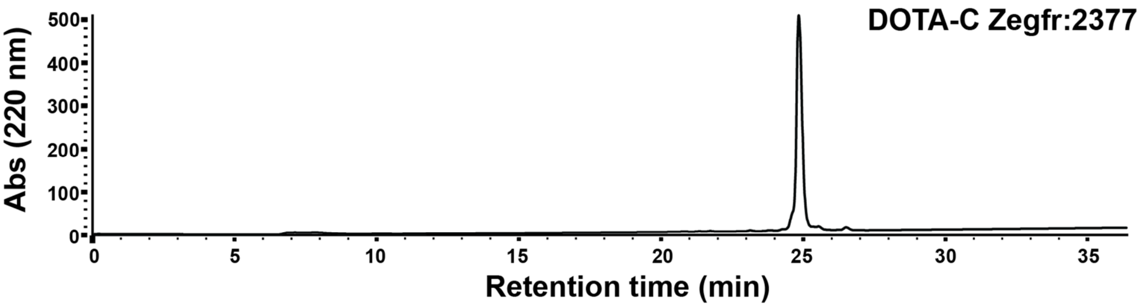

Supplementary Figure S1. Analytical RP-HPLC analysis of purified DOTA-$Z_{EGFR:2377}$. The absorbance was measured at 220 nm.

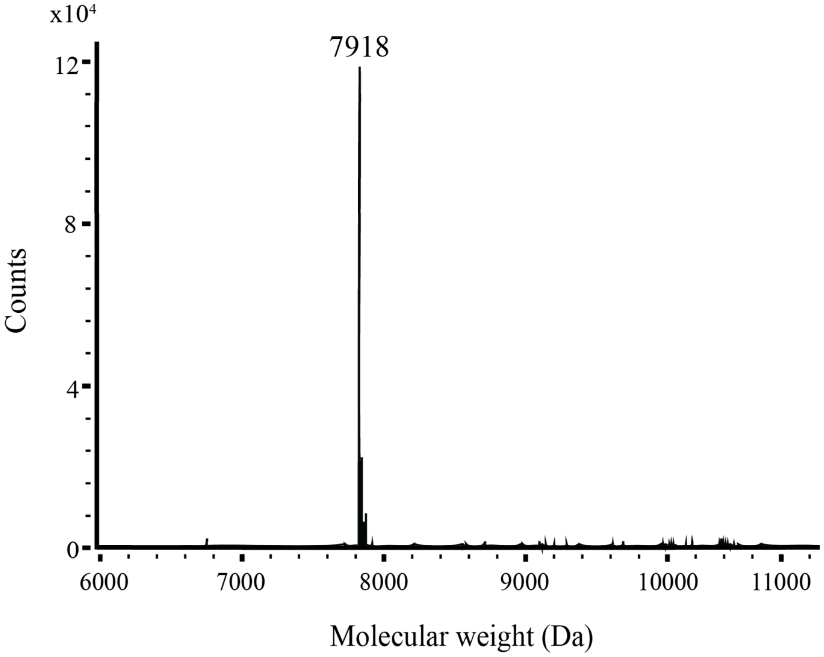

Supplementary Figure S2. Mass spectrum of purified DOTA-$Z_{EGFR:2377}$.

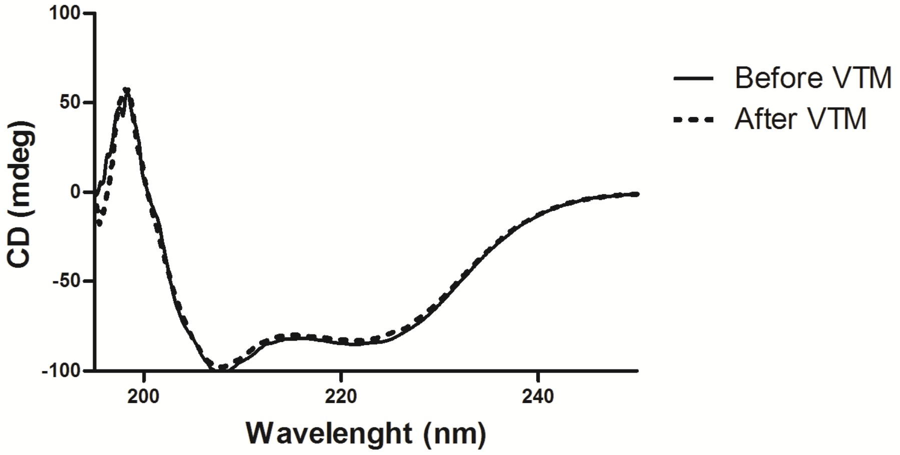

Supplementary Figure S3. Overlay of circular dichroism spectra from 195- 250 nm for DOTA-$Z_{EGFR:2377}$ before (solid line) and after (dashed line) heating from 20-90°C.

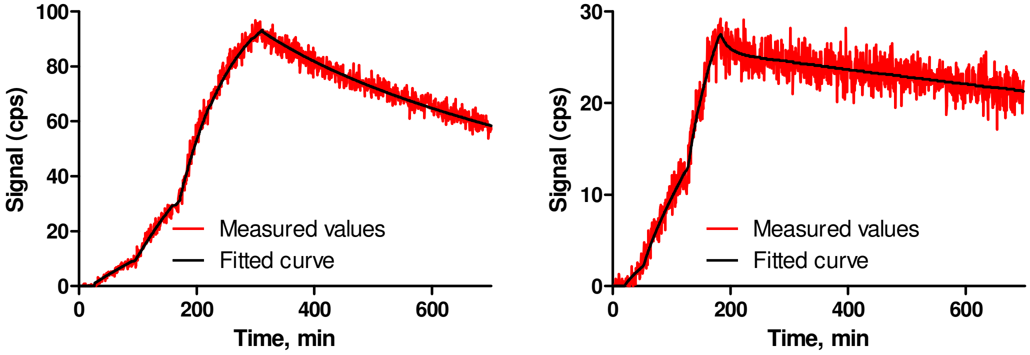

Supplementary Figure S4. Representative sensorgrams of the LigandTracer measurement to determine the kinetics and affinity of ^{111}In- DOTA-$Z_{EGFR:2377}$ (left) and ^{57}Co-DOTA-$Z_{EGFR:2377}$ (right) to EGFR on A431 cells.

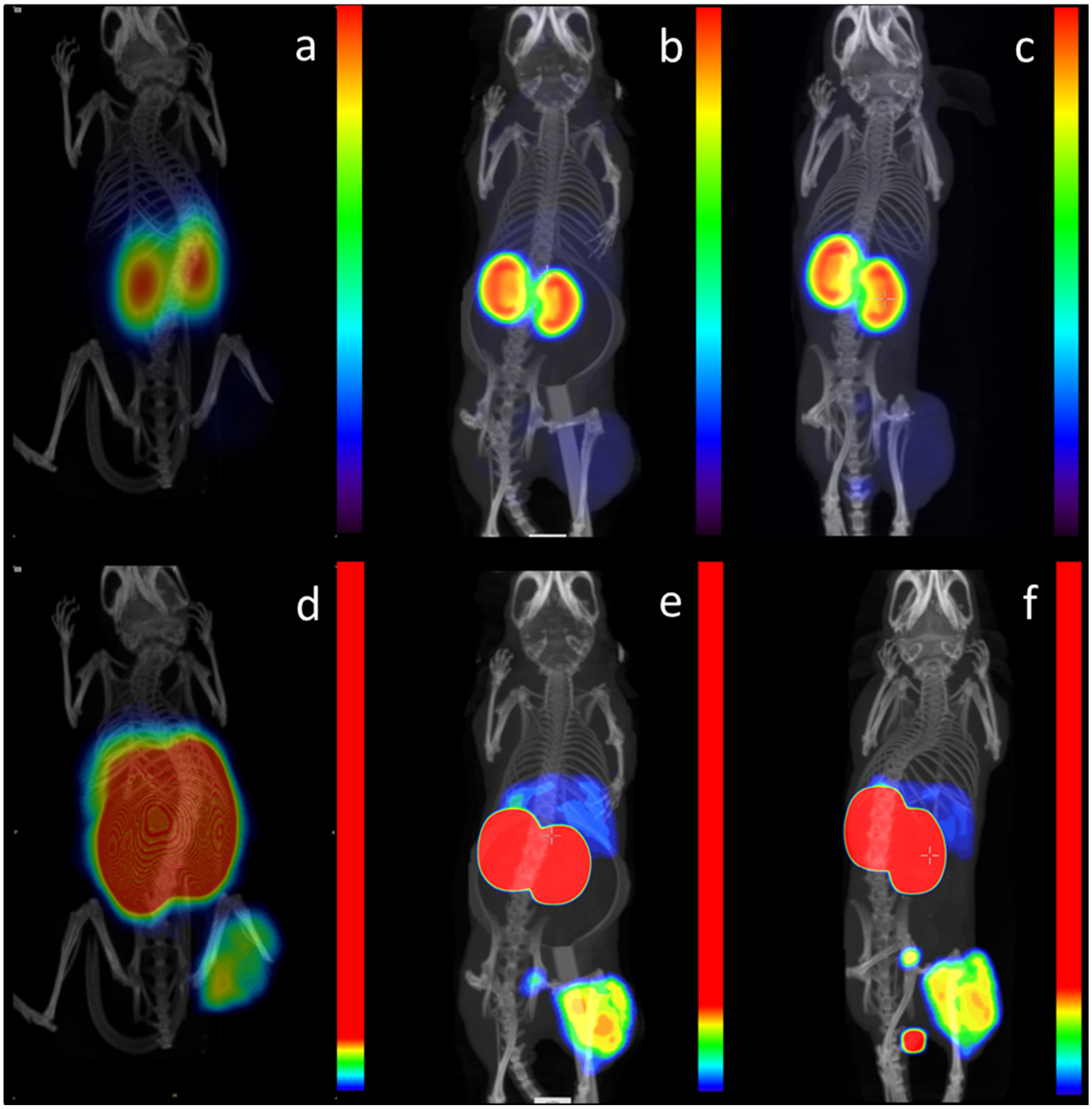

Supplementary Figure S5. PET/CT imaging using ^{68}Ga-DOTA-Z$_{EGFR:2377}$ (a and d) and microSPECT/CT using ^{57}Co-DOTA-Z$_{EGFR:2377}$ at 3 h (b and e) and 24 h (c and f) after injection of mice bearing EGFR-expressing A431 xenografts. The relative colour scales were normalized to the highest activity in images a, b and c, and then adjusted to provide first red pixel in tumours in images d, e and f (8, 13 and 17% of the full scale, respectively).

Supplementary Table S1. Radiochemical yields and specific activities of imaging probes used in this study.

	Yield, decay corrected; %	Max. specific activity at the end of synthesis; MBq/µg (GBq/µmol)
^{57}Co-DOTA-$Z_{EGFR:2377}$	> 99%	0.30 (2.34)
^{55}Co-DOTA-$Z_{EGFR:2377}$	> 99%	0.98 (7.05)
^{68}Ga-DOTA-$Z_{EGFR:2377}$	98.9±0.5%	2.05 (16.3)

Supplementary Table S2. Long-lived positron-emitting radionuclides. Data are taken from

Tolmachev, V. & Stone-Elander S. Radiolabelled proteins for positron emission tomography: Pros and cons of labelling methods. *Biochim. Biophys. Acta.* **1800**, 487-510 (2010).

Nuclide	Half-life, hour	Mode of decay	Principal photon emissions, keV (abundance in %)
^{55}Co	17.5	β^+ 76 %<br>EC 24 %	**511(152%),** 477(20.2 %), 931(75 %), 1317(7.1%), 1408(16.9%)
^{64}Cu	12.7	β^+ 18<br>β^- 37%<br>EC 24 %	**511(36%),** 1346(0.5%)
^{76}Br	16.2	β^+ 54%<br>EC 46%	**511(108%),** 559(74%), 657(15.9%),1216(8.8%), 1854(14.7%), 2391(4.7%), 2792(5.6%), 2950.5(7.4%)
^{86}Y	14.7	β^+ 33 %<br>EC 67 %	**511(66%),** 443(16.9%), 628(32.6%), 646(9.2%), 703(15.4%), 778(22.4%), 1077(82.5%), 1153(30.5%), 1854(17.2%), 1920(20.8%)
^{89}Zr	78.4	β^+ 23%<br>EC 77%	**511(46%),** 909(100%)
^{124}I	100.2	β^+ 23%<br>EC 77%	**511(46%),** 603(61%), 723(9.96%), 1691(10.4%)
